# Precise 3D geometric phenotyping and phenotype interaction network construction of maize kernels

**DOI:** 10.3389/fpls.2025.1438594

**Published:** 2025-04-08

**Authors:** Shuaihao Zhao, Guanmin Huang, Si Yang, Chuanyu Wang, Juan Wang, Yanxin Zhao, Minxiao Duan, Ying Zhang, Xinyu Guo

**Affiliations:** ^1^ Information Technology Research Center, Beijing Academy of Agriculture and Forestry Sciences, Beijing, China; ^2^ Beijing Key Laboratory of Digital Plant, China National Engineering Research Center for Information Technology in Agriculture, Beijing, China; ^3^ Beijing Key Laboratory of Maize DNA (DeoxyriboNucleic Acid) Fingerprinting and Molecular Breeding, Maize Research Center, Beijing Academy of Agriculture and Forestry Sciences, Beijing, China

**Keywords:** maize kernels, plant phenomics, 3D point cloud model, phenotypic analysis, phenome interaction network

## Abstract

Accurate identification of maize kernel morphology is crucial for breeding and quality improvement. Traditional manual methods are limited in dealing with complex structures and cannot fully capture kernel characteristics from a phenome perspective. To address this, our study aims to develop a high-throughput 3D phenotypic analysis method for maize kernels using Micro-CT-based point cloud data, thereby enhancing both accuracy and efficiency. We introduced new phenotypic indicators and developed a kernel phenome interaction network to better characterize the diversity and variability of kernel traits. Using a natural population of maize, high-resolution 2D slice data from Micro-CT scans were converted into 3D point cloud models for detailed analysis. This process led to the proposal of five new indicators, such as the endosperm density uniformity index (ENDUI) and endosperm integrity index (ENII), and the construction of their corresponding phenome interaction network. The study identified 27 3D morphological feature parameters, significantly improving the accuracy of kernel phenotypic analysis. These new indicators enable a more comprehensive evaluation of trait differences between subgroups. Results show that ENDUI and ENII are central to the phenome interaction networks, revealing synergistic relationships and environmental adaptation strategies during kernel growth. Additionally, it was found that length traits significantly impact the volumes of the embryo and endosperm, with linear regression coefficients of 0.599 and 0.502, respectively. This study not only advances maize kernel morphology research but also offers a novel method for phenotypic analysis. By enriching the phenotypic diversity of maize kernels, it contributes to breeding programs and grain processing improvements, ultimately enhancing the quality, and utilization value of maize kernels.

## 1 Introduction

Maize (*Zea mays*. L) kernels are one of the primary sources of food for humans (Li et al., 2021; Liu et al., 2017). The morphological characteristics of maize kernels directly determines their nutritional composition and processing quality, which are closely related to their food functionality and industrial value (Liu et al., 2008). Morphological characteristics of kernels, such as size, shape, compactness, internal tissue differentiation, and the distribution of starch and proteins, not only affect their storage quality and nutritional value but also determine their processing suitability and sensory quality (Ignjatovic-Micic et al., 2015). Therefore, an in-depth analysis of the developmental patterns of kernel morphology and its functional relationships is of significant theoretical and practical importance for improving the comprehensive yield and utilization value of maize.

In recent years, advances in analytical techniques have led to significant progress in the study of kernel morphology. For instance, methods for measuring maize kernel volume have evolved through various stages, including the displacement method (alcohol displacement) (Sala et al., 2007; Zhang et al., 2016; Li et al., 2022), mathematical calculations (Singh et al., 2004; Barnwal et al., 2012), and Micro-CT measurement (Zhao et al., 2021; Gustin et al., 2013; Guelpa et al., 2016; Li et al., 2023). These advancements have greatly enhanced research throughput. The application of three-dimensional (3D) tomography technology, specifically Micro-CT (micro-focus X-ray computed tomography), provides a new approach for non-destructive and high-precision acquisition of the internal and external microstructures of maize kernels. This technology can reconstruct 3D images by measuring the varying absorption rates of X-rays by plant tissues (Du et al., 2019), revealing hidden internal structural changes. This significantly expands the measurable dimensions of maize kernel phenotypes. For example, Hou et al. (2019) utilized CT to obtain various phenotypic indicators of maize kernels and discovered that the breakage rate of kernels is primarily influenced by factors such as kernel specific surface area and subcutaneous cavity volume. This study provided new insights into the relationship between kernel quality and morphological structure. Similarly, Orina et al. (2017) used Micro-CT technology to perform time-series analyses of the dynamic effects of fungal infection on the internal structure of kernels.

Despite significant progress in the analysis of kernel morphology using advanced techniques such as Micro-CT (Rousseau et al., 2015; Du et al., 2019; Zhao et al., 2021; Yin et al., 2021; Liao et al., 2022; Li et al., 2023), several challenges remain. Firstly, while the number of three-dimensional phenotypic indicators has increased, they are often limited to individual features such as volume and density (Gustin et al., 2013; Hou et al., 2019). There is a lack of systematic quantitative characterization of overall morphological variation. As a complex geometric system, the kernel’s surface features and internal tissue arrangements, in addition to basic size and shape, are closely related to its functionality. Therefore, it is necessary to establish an indicator system that can comprehensively represent kernel morphological differences. Secondly, high-throughput phenotypic identification of large-scale germplasm resources still faces technical bottlenecks (Yang et al., 2020; Song et al., 2021). Although Micro-CT can non-destructively obtain three-dimensional data, issues such as long scanning times and limited sample sizes restrict its application in genetic diversity studies. Currently, there is a lack of methods to quickly and efficiently obtain three-dimensional morphological phenotypes, leading to a scarcity of phenotypic data for germplasm resources and making it difficult to identify conformity indicators that reflect kernel morphological variation.

Based on an analysis of the current state of kernel morphological structure research, this study aims to use a maize association analysis population (Yang et al., 2011) to expand kernel phenotypic variation. Utilizing Micro-CT as a technical tool, this study aims to establish a high-throughput method for rapid analysis of three-dimensional kernel morphological phenotypes and to construct an indicator system that can comprehensively characterize kernel morphological variation. This will lay a solid foundation for the genetic analysis of kernel structure development regulation. The specific research objectives are as follows: 1) transform traditional Micro-CT image data into three-dimensional point cloud data to enhance the visualization and quantification of kernel phenotypes; 2) based on point cloud data, develop an indicator system that can describe the overall morphological characteristics of kernels from multiple angles and levels, fully characterizing the morphological differences among various germplasm resources; 3) using the aforementioned phenotypic analysis methods and indicator systems, establish a high-precision, high-throughput three-dimensional phenotypic database for a large-scale natural maize population. This database will provide a high-quality data source for subsequent genetic analyses, such as genome-wide association studies of kernel morphological structure.

By achieving these research objectives, we can systematically analyze the developmental patterns of maize kernel morphology and their intrinsic relationships with quality, providing theoretical guidance for designing and breeding high-quality, high-yield new varieties. Additionally, it will help accelerate the identification of key regulatory genes and the creation of related molecular markers, providing new tools for molecular design breeding. This is of great significance for enhancing the comprehensive yield and utilization value of maize.

## 2 Materials and methods

### 2.1 Experimental materials

The experimental materials used in this study included 288 maize association analysis population, sourced from a population planted in 2018 at the Sanya base of the Beijing Academy of Agriculture and Forestry Sciences in Hainan. These materials comprised 57 samples from the Mixed subgroup (Mixed), 84 from the NSS (Non-Stiff Stalk) subgroup, 21 from the SS (Stiff Stalk) subgroup, and 126 from the TST (Tropical-Subtropical) subgroup. Maize was planted with an equal row spacing of 60 cm and a planting density of 67,500 plants ha^-1^. To ensure the genetic stability of the kernel samples, artificial bagging and self-pollination were performed on each variety before silking. After harvesting the ears, 3 ears were randomly selected from each material, and 3 kernels of similar size and fullness were taken from the middle of each ear for subsequent phenotypic data acquisition and analysis.

### 2.2 Kernel scanning and reconstruction

**Figure 1:**
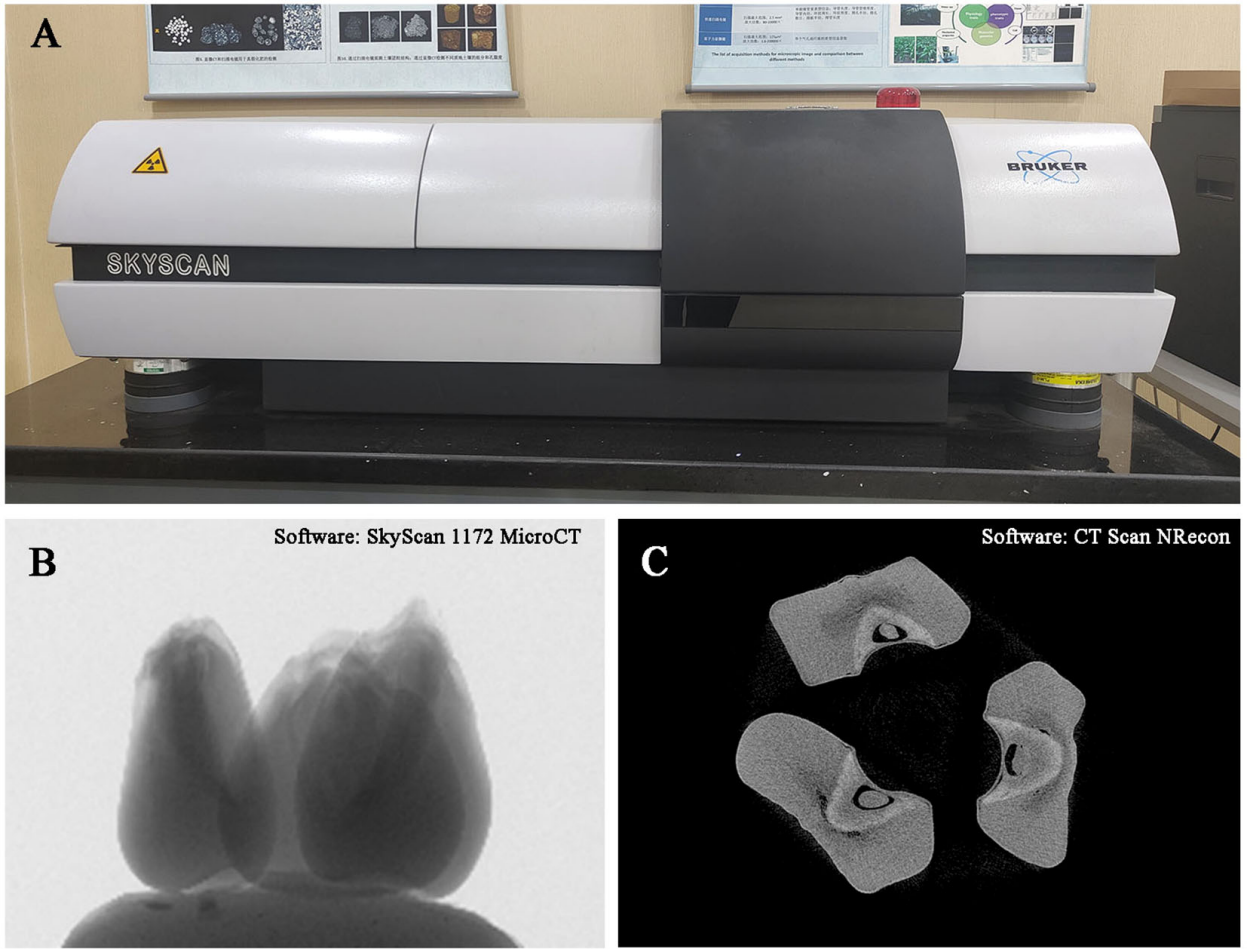
CT scanning instrument **(A)** and kernel reconstruction **(B, C)**.

Kernel CT phenotypic data were obtained using a 1172 model Micro-CT device (Bruker Corporation, Billerica, MA, USA, **Figure 1A**). During scanning, the kernels were fixed on foam boards and placed inside the CT machine. The scanning was set to 2K mode (2000 × 1332 pixels), with a resolution of 13.55 μm. The scanning voltage was 40 kV, and the current was 250 μA. The system performed continuous scanning of the samples at 0.4° intervals for a total of 180°. Subsequently, the raw CT scan images were reconstructed using CT Scan NRecon 1.6.9.4 (Micro Photonics Inc, Allentown, PA, USA, **Figures 1B, C**) software, resulting in a series of reconstructed virtual kernel cross-sectional images with a resolution of 2000 × 2000 pixels in 8-bit BMP format.

### 2.3 Kernel phenotypic data analysis

The CornSeger software (Li et al., 2023) was used to extract 13 three-dimensional kernel indicators. CornSeger outputs three folders containing kernel CT cross-sectional images, cavity segmentation results, and endosperm and embryo segmentation results, saved in.nii format to a specified path. The segmentation results can be viewed using ITK-SNAP (Yushkevich et al., 2016). To convert CT data into point cloud data, the Open3D library (an open-source 3D data processing library) in Python was used. The computing environment was as follows: CPU Intel Core i7-13700, Windows 11 operating system, NVIDIA GeForce RTX 3060 Ti graphics card, Python 3.9.0, and PyCharm 2021.3.2.

### 2.4 Kernel phenotypic data analysis

This study evaluated kernel structure from five perspectives, primarily including endosperm nutrient density, endosperm integrity, embryo volume-to-surface area ratio, kernel coat tightness, and endosperm density uniformity. The specific calculation formulas are as follows:

Endosperm Nutrient Density Index (ENDI): This index quantifies the spatial distribution density of nutrients in endosperm tissue. Higher ENDI values indicate greater nutrient content per unit volume, closely associated with the efficient accumulation of macromolecules such as carbohydrates and proteins. From a physiological perspective, this parameter reflects the efficiency of nutrient translocation and deposition, subsequently influencing nutrient mobilization capacity during germination and seedling establishment rate. The calculation formula is (Equation 1):


(1) $$ENDI=\frac{ENV}{ENL\;*\;ENW\;*\;ENT}$$

where ENV is endosperm volume, ENL is endosperm length, ENW is endosperm width, ENT is endosperm thickness.

Endosperm Integrity Index (ENII): This index characterizes the structural integrity of endosperm tissue. An ENII value approaching 1 indicates dense endosperm matrix structure and tight cellular arrangement, features that promote mechanical strength and physiological activity maintenance. Intact endosperm structure not only ensures storage compound stability but also regulates germination metabolism through modulation of water absorption and enzymatic reaction rates. The calculation formula is (Equation 2):


(2) $$ENII=1\;-\;\frac{CV}{\;ENV}$$

where CV is cavity volume, ENV is endosperm volume.

Embryo Volume-Surface Ratio (EMVSR): This index reflects the relationship between embryo morphological characteristics and functional potential. Higher EMVSR typically indicates greater nutrient storage capacity and more developed organ primordia, directly correlating with embryo metabolic activity and morphogenetic capability. From a developmental biology perspective, this parameter can predict the differentiation potential of photosynthetic organs and the development capacity of vegetative organs during seedling stage. The calculation formula is (Equation 3):


(3) $$EMVSR=\frac{EMV}{EMS}$$

where EMV is embryo volume, EMS is embryo surface area.

Seed Coat Tightness Index (SCTI): This index delineates the structural coordination between seed coat and internal tissues. SCTI values reflect potential stress resistance levels, with higher values indicating enhanced physical barrier function of the seed coat. This encompasses not only mechanical protection but also physiological functions in regulating gas exchange and water permeability, thereby influencing the spatiotemporal progression of seed dormancy and germination. The calculation formula is (Equation 4):


(4) $$SCTI=\frac{KV}{KSP\;*\;KV}$$

where KV is kernel volume, KSP is kernel sphericity.

Endosperm Density Uniformity Index (ENDUI): This index assesses the spatial uniformity of material distribution in endosperm tissue. ENDUI values approaching 0 indicate more uniform nutrient distribution, a characteristic associated with cell division/differentiation synchronicity and transport efficiency. Uniform material distribution helps maintain stable metabolic activity, ensuring continuous nutrient supply during germination processes. The calculation formula is (Equation 5):


(5) $$ENDUI=1\;-\;\frac{ENS}{ENL\;*\;ENW\;*\;ENT}$$

where ENS is endosperm surface area, ENL is endosperm length, ENW is endosperm width, ENT is endosperm thickness.

### 2.5 Data analysis

During the preliminary data processing, 27 kernel phenotypic traits data were organized using Microsoft Excel 2016. Subsequently, descriptive statistical analysis was performed using R 4.3.1 software. The pastecs package (version 1.4.2) was used to calculate the median, minimum, maximum, mean, coefficient of variation, skewness, and kurtosis for each phenotype.

For correlation analysis among traits, NetCoMi package (version 1.1.0) (Peschel et al., 2021) was employed to construct the correlation network. In the data preprocessing stage, missing values were detected and removed, followed by centered log-ratio (CLR) transformation for data standardization to eliminate compositional effects. Network construction was based on Pearson correlation coefficients as association measures, with a correlation coefficient threshold of |r|≥0.3 to screen significant correlations. Multiple replacement methods were used to handle zero values, and network sparsification was achieved through threshold methods. Network analysis employed the Fast Greedy clustering algorithm for module division, which identifies closely related trait groups by optimizing modularity. Node sizes were calculated based on eigenvector centrality to reflect the importance of each trait in the network. For network visualization, parameters such as node size, transparency, connection curvature, and node repulsion were finely adjusted to ensure network clarity and readability. Positive correlations were represented in green, negative correlations in red, different colored nodes represented different functional modules, and node size reflected the centrality of that trait in the overall network. This network analysis method not only visually displayed the strength and direction of trait relationships but also identified trait groups with similar functions or regulatory relationships through clustering, providing important analytical basis for understanding complex trait associations.

Analysis of variance (ANOVA) among subgroups was performed using the agricolae package (version 1.3.7), with multiple comparisons conducted using HSD.test (P < 0.05). This step helped determine significant differences between subgroups and further clarified which subgroups showed significant differences through multiple comparison methods.

## 3 Results

### 3.1 Construction of a kernel 3D phenotyping system based on point cloud data

**Figure 2:**
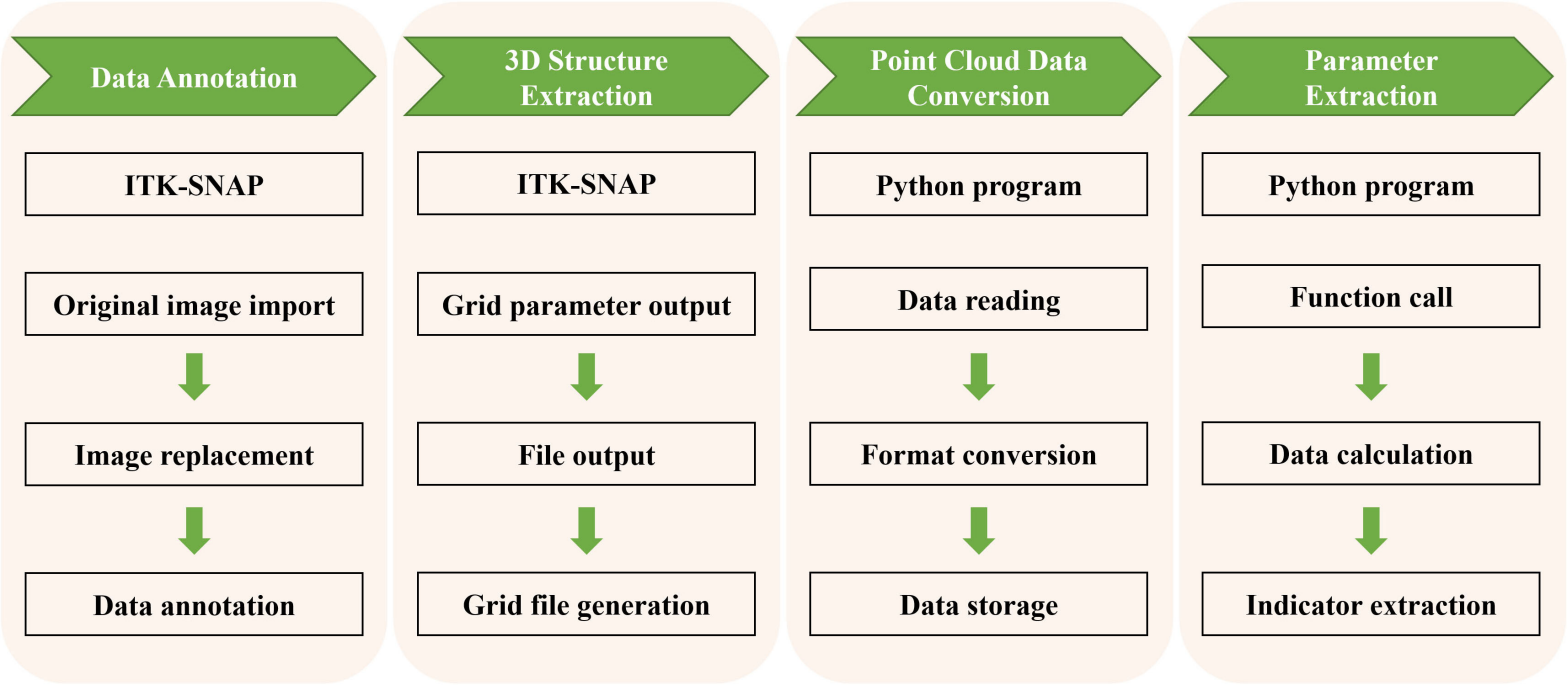
Workflow of maize kernel data annotation and parameter extraction.

**Figure 3:**
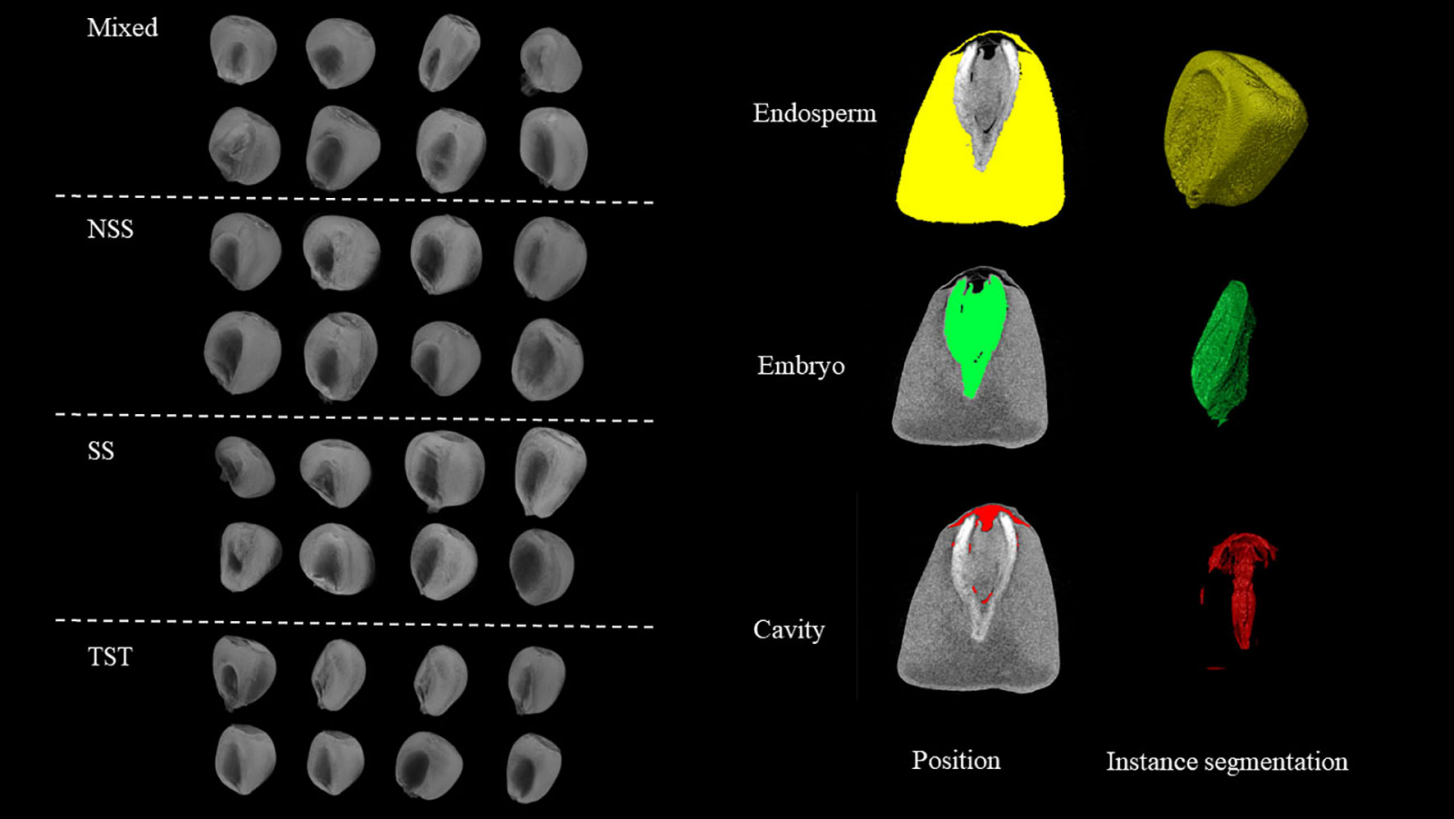
Maize kernel overview and instance segmentation.

In this study, we developed a 3D phenotyping system based on point cloud data, which allows for high-throughput, non-destructive, and precise extraction of quantitative parameters related to the spatial relationships among the embryo, endosperm, and pericarp of maize kernels. This process mainly involves four parts: data import and annotation, 3D structure extraction, point cloud data conversion, and high-throughput geometric parameter extraction (**Figure 2**). Additionally, by comparing the spatial positions of the embryo point cloud data with the kernel pericarp surface point cloud data, we calculated the Euclidean distance from each point on the embryo surface to the pericarp surface. This 3D phenotyping system enables high-throughput, non-destructive, and precise phenotypic analysis of maize kernel structures (**Figure 3**), overcoming the subjectivity and inefficiency of traditional manual measurements. The extracted quantitative parameters not only assess kernel quality but, more importantly, provide critical scientific evidence for understanding the correlation between embryo position and kernel traits such as development and germination.

### 3.2 Descriptive statistics of phenotypic traits

After conducting a comprehensive descriptive statistical analysis of 27 maize kernel traits (**Supplementary Table S1**), we revealed the diverse distribution characteristics and variability of these traits. For most traits, the mean and median were close, such as the mean of ENDI (0.03) and its median (0.03), indicating a relatively symmetrical distribution, approximating a normal distribution. Similarly, ENL (mean 0.31, median 0.31) and EMW (mean 0.37, median 0.68) also showed symmetrical distributions (**Table 1**). However, certain traits exhibited significant skewness and kurtosis. KSur showed a pronounced right-skewed distribution with a mean of 267.59, a median of 252.12, skewness of 1.38, and kurtosis of 4.81, indicating data concentrated at higher values with significant outliers. Similarly, KSSA and CV also displayed right-skewed distributions, with skewness values of 1.11 and 1.50 and kurtosis values of 3.80 and 5.19, respectively, indicating more outliers in the higher value range. Conversely, ENP and ENII exhibited left-skewed distributions (**Table 1**). ENP had a skewness of -0.72 and kurtosis of -2.51, while ENII had a skewness of -1.50 and kurtosis of 2.45, indicating more data points in the lower value range. Notably, ENII had the lowest coefficient of variation (0.004), with data highly concentrated around a value close to 1.00, showing significant consistency. For kurtosis analysis, the high kurtosis values of KSur and CV (4.81 and 5.19, respectively) suggested steep distributions with more extreme values, whereas the low kurtosis values of Hgw and SCTI (-0.07 and -0.019, respectively) indicated flatter distributions with fewer extreme values (**Table 1**). Additionally, the coefficient of variation analysis showed that the coefficients of variation for different traits ranged from 0.004 (ENII) to 0.86 (CV), reflecting differences in relative variability among traits. For example, CP had a coefficient of variation of 0.74, a mean of 4.13E-03, and a median of 3.17E-03, indicating high relative variability and a right-skewed distribution (skewness 1.17, kurtosis 4.00). These descriptive statistical results elucidate the complex distribution characteristics of maize kernel traits under genetic and environmental influences (**Table 1**). Most traits displayed symmetrical distributions with moderate variability, but certain traits, such as KSur, CV, and ENII, exhibited significant skewness and kurtosis, indicating data concentrated around extreme values or high consistency (**Table 1**).

**Table 1:** Descriptive statistics of maize kernel phenotypic characteristics.

Trait	Median	Min	Max	Mean	CV	Skewness	Kurtosis
KS	0.22	0.13	0.38	0.22	0.19	0.60	2.09
KV	197.05	33.58	421.35	203.39	0.21	0.39	1.36
KSur	252.12	83.85	547.39	267.59	0.23	1.38	4.81
KSSA	1.26	0.96	2.03	1.31	0.17	1.11	3.80
KSP	0.36	0.19	0.63	0.36	0.24	0.48	1.67
EMV	29.63	4.59	60.60	30.12	0.24	0.65	2.25
EMS	94.81	21.69	184.67	97.84	0.20	0.57	1.97
EMP	0.15	0.09	0.24	0.15	0.17	0.60	2.07
ENV	167.85	28.94	389.70	172.29	0.22	0.59	2.06
ENS	238.43	57.16	509.77	242.52	0.18	0.67	2.33
ENP	1.57	1.43	1.64	1.56	0.02	-0.72	-2.51
CV	0.67	4.55E-03	3.79	0.93	0.86	1.50	5.19
CP	3.17E-03	2.72E-05	0.01	4.13E-03	0.74	1.17	4.00
Hgw	24.37	10.43	41.68	24.50	0.19	-0.02	-0.07
MiD	12.03	5.44	18.54	11.98	0.21	-0.1	-0.4
MaD	33.90	22.17	44.00	33.78	0.10	-0.25	0.46
EML	19.21	8.95	25.61	19.21	0.12	-0.26	1.36
EMW	11.67	5.46	17.97	11.84	0.15	0.37	0.68
EMT	7.38	4.94	10.96	7.51	0.13	0.44	0.29
ENL	21.64	13.39	29.54	21.87	0.12	0.31	0.41
ENW	20.68	8.51	27.31	20.72	0.12	-0.37	1.32
ENT	12.15	7.19	19.41	12.42	0.16	0.52	0.18
ENDI	0.03	0.02	0.04	0.03	0.09	-4.88E-04	-2.18E-01
ENII	1.00	0.98	1.00	0.99	0.004	-1.50	2.45
EMVSR	0.31	0.21	0.40	0.31	0.10	-5.62E-02	-5.87E-02
SCTI	2.78	1.60	5.36	2.91	0.25	0.61	-1.90E-02
ENDUI	0.96	0.93	0.97	0.96	0.005	-7.99E-01	2.23

**Figure 4:**
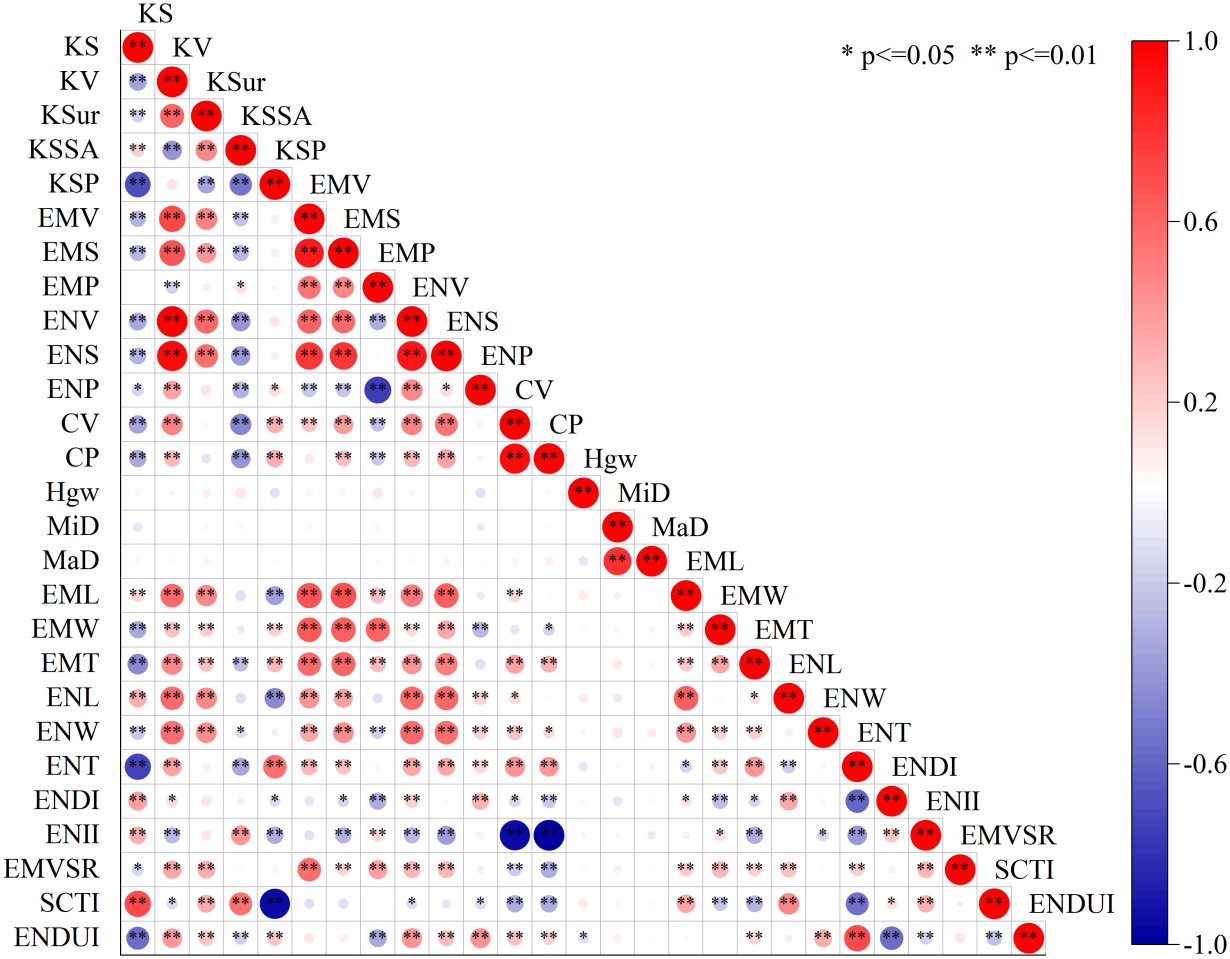
Heatmap of correlations among kernel phenotypic parameters.

Correlation analysis revealed complex relationships among the 27 phenotypic traits (**Figure 4**). Among volume-related traits, KV showed the strongest positive correlation with ENV (r=0.99, p ≤ 0.01), while EMV exhibited a high positive correlation with embryo surface area EMS (r=0.90, p ≤ 0.01). Regarding morphological characteristics, KS demonstrated a significant negative correlation with KSP (r=-0.70, p ≤ 0.01), while showing a significant positive correlation with SCTI (r=0.71, p ≤ 0.01). Among structural integrity indicators, CV displayed a strong positive correlation with CP (r=0.96, p ≤ 0.01), while ENII exhibited a perfect negative correlation with CP (r=-1.00, p ≤ 0.01). Furthermore, significant correlations were observed among dimensional parameters, with EMV showing significant positive correlations with its three-dimensional parameters (EML, EMW, EMT) (r values ranging from 0.58 to 0.67, p ≤ 0.01). These correlation patterns reveal the intrinsic relationships in maize kernel structural development, providing a foundation for further optimization of phenotypic evaluation systems.

### 3.3 Construction and analysis of phenotypic interaction network

**Figure 5:**
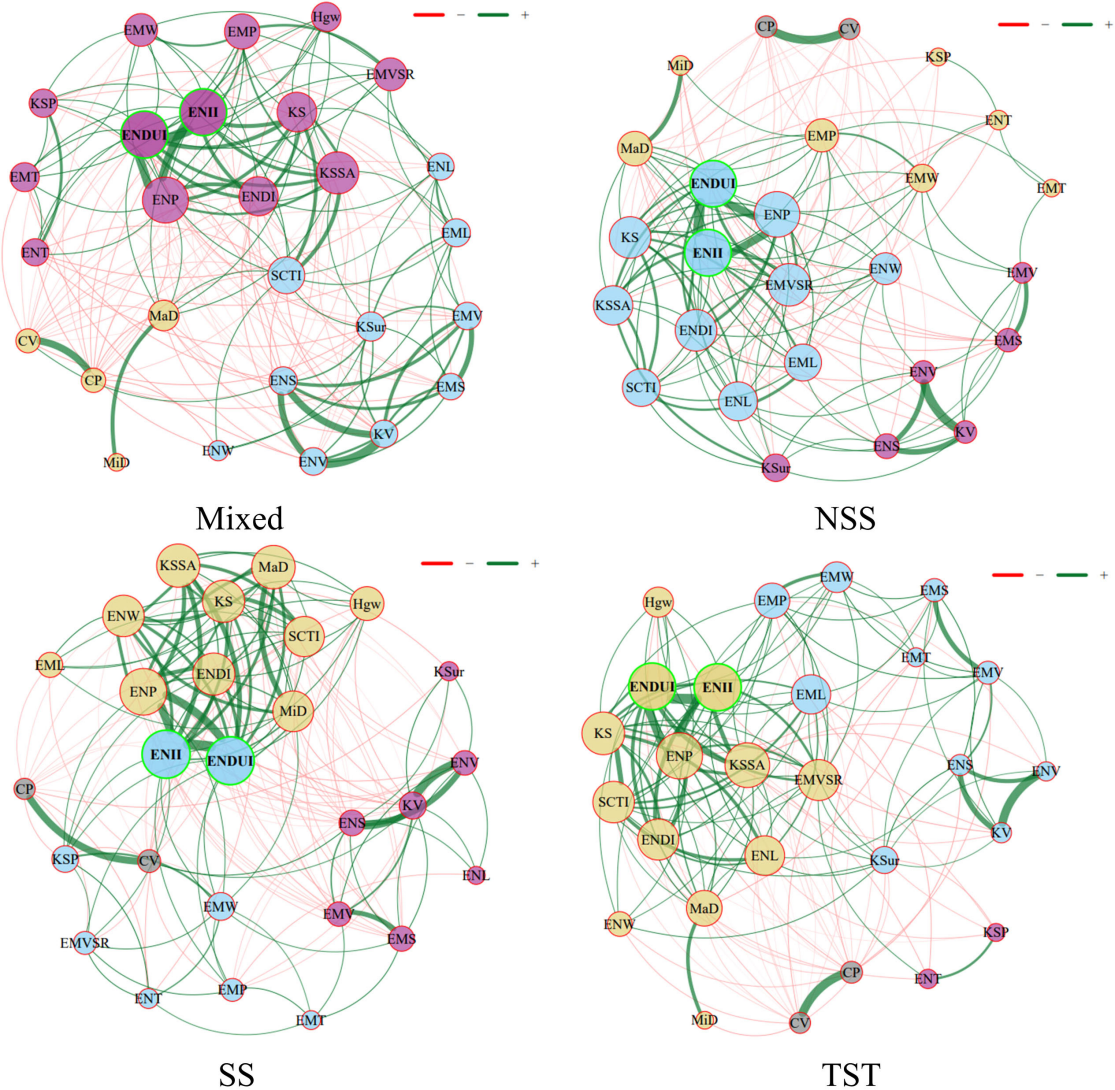
Kernel phenotypic interactome networks across different subgroups.

In this study, we constructed a phenotypic interaction network and used nodes and edges to describe the phenotypic relationships within different subgroups. In this network, nodes represent different operational taxonomic units (OTUs, i.e., phenotypes), and edges represent the associations between these operational taxonomic units, where green edges indicate positive correlations and red edges indicate negative correlations (**Figure 5**). Additionally, the thickness of the edges represents the strength of the association, the thicker the edge, the stronger the association. The results showed that in different subgroups, ENII, ENDU, and ENP were high-degree nodes (**Figure 5**). These nodes occupied central positions in the network with high connectivity, indicating that they play crucial roles in different subgroups while maintaining important balance. Moreover, the number of green edges was significantly higher than the number of red edges across different subgroups, indicating that these OTUs primarily engage in synergistic interactions (**Figure 5**). This also suggests that these phenotypic traits tend to form mutualistic phenotypic networks within different subgroups, enhancing intra-group stability and functionality. The nodes in the Mixed and SS subgroups were more concentrated, forming tight network structures, indicating a more stable and unified phenotypic network structure under these conditions. In contrast, under the NSS and TST conditions, the nodes were more dispersed, showing a more complex and heterogeneous subgroup phenotypic structure (**Figure 5**). This distribution difference may reflect the growth adaptation strategies of different subgroups: tight cooperation versus dispersed and diverse growth strategies. Although certain nodes like Hgw and KSur had lower connectivity under Mixed conditions, they might play key ecological functions in specific environments (**Figure 5**). The negative correlations shown by the red edges may reflect competitive relationships between different phenotypes within the subgroups, which could affect the diversity and stability of the subgroups.

### 3.4 Analysis of differences in kernel phenotypic traits across subgroups

After an in-depth analysis of kernel phenotypic traits across four different subgroups (mixed subgroup, non-stiff stalk subgroup, stiff stalk subgroup, and tropical/subtropical subgroup), five traits were found to have significant differences among the subgroups (**Table 2**), including surface area (P = 2.19E-04), specific surface area (P = 7.86E-04), 100-kernel weight (P = 3.18E-02), embryo width (P = 2.07E-02), and the ratio of embryo volume to surface area (P = 3.69E-02). The stiff stalk subgroup and tropical/subtropical subgroup had smaller surface areas, with the stiff stalk subgroup having a 10.0% and 9.6% lower surface area than the mixed subgroup and non-stiff stalk subgroup, respectively, while the tropical/subtropical subgroup had an 11.8% lower surface area. In terms of specific surface area, the tropical/subtropical subgroup was 8.8% and 9.5% lower than the mixed subgroup and non-stiff stalk subgroup, respectively, and 8.1% lower than the stiff stalk subgroup (**Table 2**). For 100-kernel weight, the non-stiff stalk subgroup was 8.1% higher than the mixed subgroup, 7.1% higher than the tropical/subtropical subgroup, and 5.7% higher than the stiff stalk subgroup (**Table 2**). Regarding embryo width, the non-stiff stalk subgroup was 6.2% higher than the tropical/subtropical subgroup, while the mixed subgroup and stiff stalk subgroup were 5.9% and 3.1% higher than the tropical/subtropical subgroup, respectively (**Table 2**). For the ratio of embryo volume to surface area, the tropical/subtropical subgroup was 3.2% lower than the non-stiff stalk subgroup and mixed subgroup. Additionally, although other traits, such as kernel length, kernel width, embryo length, and embryo height, also showed differences across subgroups, these differences were not significant, indicating a more uniform distribution of these traits across subgroups (**Table 2**).

**Table 2:** Analysis of differences in 27 maize kernel traits across four subgroups.

Trait	P-value	Mixed	NSS	SS	TST
KS	2.81E-01	0.22 ± 0.04a	0.22 ± 0.04a	0.22 ± 0.03a	0.23 ± 0.04a
KV	5.04E-01	204.59 ± 46.62a	207.23 ± 43.86a	194.76 ± 47.50a	201.73 ± 40.24a
KSur	2.19E-04	284.63 ± 76.94a	283.62 ± 64.96a	256.28 ± 41.07ab	251.21 ± 50.87b
KSSA	7.86E-04	1.36 ± 0.26a	1.37 ± 0.27a	1.35 ± 0.20ab	1.24 ± 0.17b
KSP	6.13E-01	0.35 ± 0.08a	0.36 ± 0.08a	0.37 ± 0.08a	0.38 ± 0.10a
EMV	8.48E-02	30.89 ± 8.30a	31.29 ± 5.58a	30.15 ± 9.74a	28.98 ± 7.06a
EMS	5.14E-01	98.45 ± 22.21a	100.10 ± 15.66a	97.67 ± 21.63a	96.08 ± 20.95a
EMP	6.25E-02	0.15 ± 0.02a	0.15 ± 0.02a	0.15 ± 0.02a	0.14 ± 0.03a
ENV	5.46E-01	172.90 ± 39.89a	175.10 ± 40.96a	163.57 ± 38.81a	171.59 ± 35.45a
ENS	6.50E-01	242.29 ± 45.00a	246.29 ± 44.05a	236.11 ± 48.61a	241.18 ± 40.30a
ENP	2.97E-01	1.56 ± 0.03a	1.56 ± 0.04a	1.56 ± 0.04a	1.57 ± 0.04a
CV	2.64E-01	0.79 ± 0.74a	0.84 ± 0.66a	1.04 ± 0.99a	1.02 ± 0.87a
CP	1.27E-01	0.003 ± 0.003a	0.004 ± 0.003a	0.004 ± 0.003a	0.005 ± 0.003a
Hgw	3.18E-02	23.81 ± 4.47b	25.73 ± 5.04a	24.34 ± 3.87ab	24.03 ± 4.66b
MiD	1.88E-01	12.07 ± 2.97a	11.75 ± 2.26a	13.07 ± 1.94a	11.91 ± 2.57a
MaD	3.53E-01	33.86 ± 4.36a	33.33 ± 3.29a	34.72 ± 2.76a	33.89 ± 3.23a
EML	4.70E-01	19.20 ± 2.36a	19.51 ± 1.81a	18.90 ± 2.58a	19.07 ± 2.34a
EMW	2.07E-02	12.16 ± 1.65ab	12.19 ± 1.56a	11.84 ± 1.39ab	11.48 ± 1.89b
EMT	6.42E-01	7.46 ± 0.85a	7.56 ± 0.89a	7.70 ± 1.26a	7.47 ± 1.05a
ENL	7.47E-01	21.54 ± 2.64a	22.06 ± 2.43a	21.43 ± 2.72a	21.96 ± 2.58a
ENW	5.75E-02	21.42 ± 2.72a	20.71 ± 2.34a	20.39 ± 1.52a	20.46 ± 2.56a
ENT	8.63E-01	12.45 ± 1.73a	12.48 ± 2.05a	12.29 ± 2.06a	12.40 ± 1.94a
ENDI	2.62E-01	0.03 ± 0.002a	0.03 ± 0.003a	0.03 ± 0.003a	0.03 ± 0.003a
ENII	1.39E-01	1.00 ± 0.004a	1.00 ± 0.004a	0.99 ± 0.005a	0.99 ± 0.004a
EMVSR	3.69E-02	0.31 ± 0.04ab	0.31 ± 0.03a	0.31 ± 0.04ab	0.30 ± 0.03b
SCTI	6.96E-01	3.00 ± 0.68a	2.97 ± 0.75a	2.84 ± 0.58a	2.85 ± 0.73a
ENDUI	2.33E-01	0.96 ± 0.004a	0.96 ± 0.004a	0.96 ± 0.004a	0.96 ± 0.005a

CV, Cavity Volume; CP, Cavity Proportion; EML, Embryo Length; E15-MarMT, Embryo Thickness; EMV, Embryo Volume; EMW, Embryo Width; EMS; Embryo Surface Area; EMP, Embryo Proportion; ENDI, Endosperm nutrient density index; ENDUI, Endosperm Density Uniformity Index; ENII, Endosperm integrity index; ENL, Endosperm Length; ENP, Endosperm Proportion; ENV, Endosperm Volume; ENS, Endosperm Surface Area; ENT, Endosperm Thickness; Hgw, 100-Kernel Weight; KSSA, Kernel Specific Surface Area; KSP, Kernel Sphericity; KSur, Kernel Surface Area; KS, Kernel Sphericity; KV, Kernel Volume; MaD, Maximum distance between the embryo and the seed coat; MiD, Minimum distance between the embryo and the seed coat; SCTI. Kernel coat tightness index. Different lowercase letters (a, b) within the same row indicate significant differences among treatments at P < 0.05 level based on Duncan's multiple range test. Values are presented as mean ± standard deviation.

### 3.5 Analysis of factors influencing embryo and endosperm volume and internal structure

Through linear regression analysis of the relationship between maize ENV and ENL, ENW, and ENT, we obtained the following equation:


ENV = 0.599 x ENL + 0.533 x ENW + 0.471 x ENT

**Figure 6:**
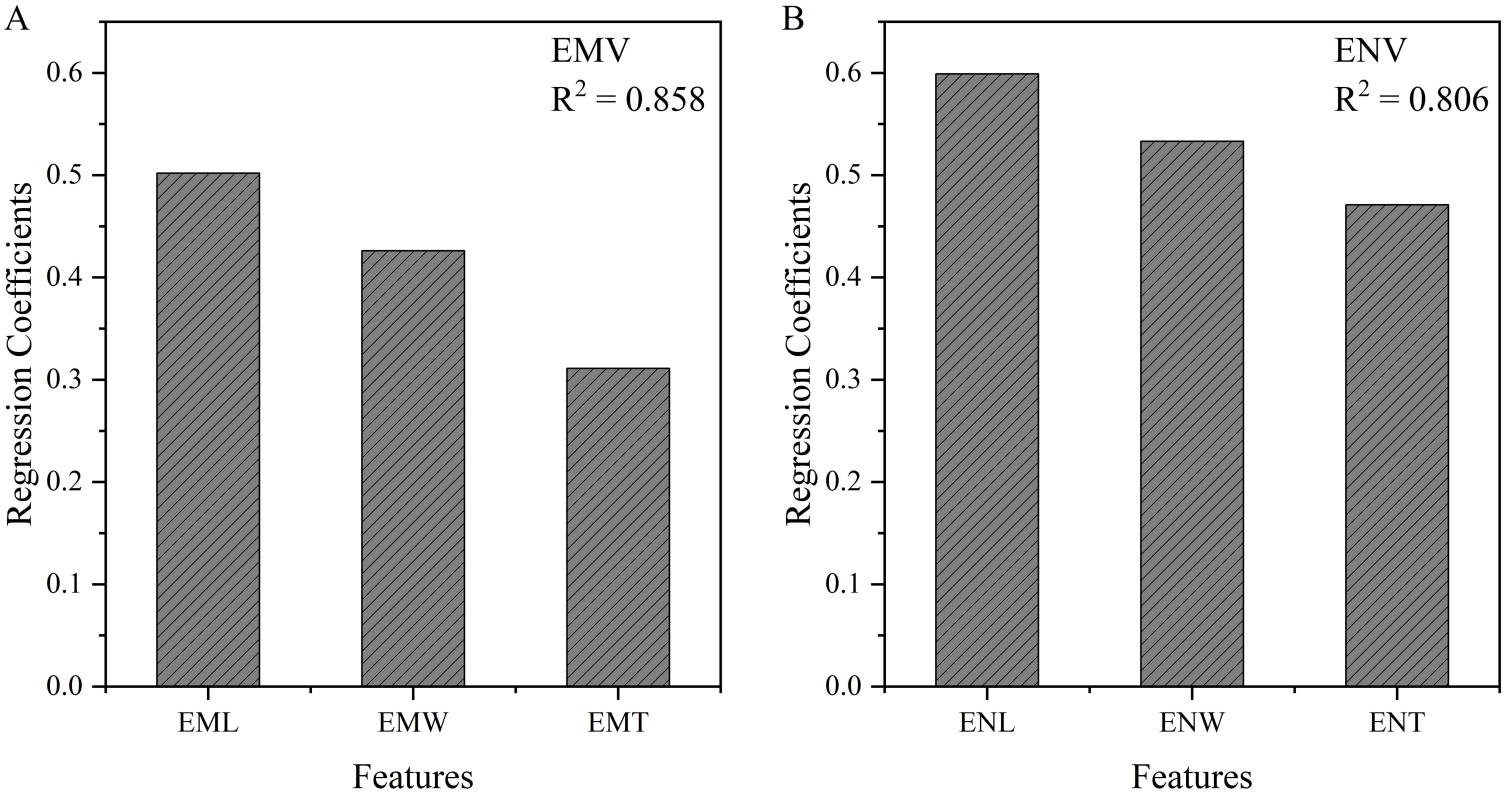
Linear regression results between embryo **(A)** and endosperm **(B)** volume and internal structure size.

From the regression coefficients, we can see that ENV is significantly influenced by ENL, ENW, and ENT (**Figure 6**), with length having the greatest impact (0.599), followed by width (0.533), and finally thickness (0.471). This indicates that during the development of maize kernels, endosperm length has the most significant effect on its overall volume, reflecting the morphological characteristics of the internal structure of maize kernels. Specifically, a longer endosperm may imply more storage material, thus having a greater impact on the nutritional value and growth potential of the kernel. Although width and thickness also play significant roles, their influence is relatively lower compared to length.

Similarly, through linear regression analysis of the relationship between EMV and EML, EMW, and EMT, the following equation was obtained:


EMV = 0.502 x EML + 0.426 x EMW + 0.311 x EMT

The analysis shows that EML contributes the most to EMV (0.502), followed by EMW (0.426), and finally EMT (0.311) (**Figure 6**). This result indicates that embryo length plays the most crucial role in determining its overall volume. Compared to the endosperm, although embryo length, width, and thickness collectively influence volume, the dominant role of length is more pronounced. This may be because the embryo requires sufficient length during kernel development to support its growth and functional realization, while width and thickness are relatively less important.

## 4 Discussion

This study proposed a point cloud-based 3D analysis method for kernels, achieving an important breakthrough in the field of 3D analysis of maize kernels (**Figures 1**, **3**). Traditional two-dimensional image analysis methods have significant limitations in dealing with complex morphological structures, such as the loss of spatial information and the inability to comprehensively reflect the internal structure of kernels (Zhao et al., 2019; Li et al., 2020; Cai et al., 2023; Li et al., 2023). In contrast, point cloud data technology, by capturing the three-dimensional geometric information of kernels (Wen et al., 2021), can accurately reproduce the morphological features of kernels, including surface texture and internal structure. Specifically, high-resolution two-dimensional slice data generated by micro-CT scanning technology was converted into a three-dimensional point cloud model, allowing the detailed recording of the surface and internal structure of each kernel (**Figure 2**). Through these point cloud data, basic morphological features such as the volume, surface area, and density of the endosperm and embryo can be calculated, and complex geometric morphological parameters, such as curvature and symmetry, can also be extracted (**Supplementary Table S1**). Compared to traditional methods, the point cloud-based 3D reconstruction method not only improves the accuracy and reliability of phenotypic analysis but also provides rich data support for the systematic study of complex morphological features (Miao et al., 2021; Wen et al., 2021). Furthermore, the processing and analysis techniques of point cloud data have high automation and high-throughput characteristics, enabling us to process a large number of samples in a short time, significantly improving the efficiency and scale of phenotypic research.

Based on the point cloud data-driven phenotypic analysis method, this study introduced several novel kernel morphological phenotypic indicators, including ENDI, ENII, EMVSR, SCTI, and ENDUI (**Figure 3**). These new indicators provide fresh perspectives for more comprehensively describing kernel morphological characteristics and internal structures. Through analysis of phenotypic data across different subgroups (Mixed, NSS, SS, TST), we found that these newly developed parameters played crucial roles in revealing intergroup differences. For instance, EMVSR was significantly lower in the TST subgroup compared to other subgroups, suggesting that the TST subgroup may possess more compact endosperm structure, which is consistent with its smaller KSur and specific surface area KSSA. Furthermore, although ENII showed no significant differences among subgroups, its values were slightly lower in SS and TST subgroups, indicating potentially simpler internal network structures of endosperm in these two subgroups. These findings provide important insights for further investigation of kernel internal structural characteristics and genetic mechanisms across different subgroups (**Figure 4**). While this study significantly enhanced the accuracy and efficiency of maize kernel morphological feature analysis through a point cloud data-based high-throughput phenotyping system, we must recognize that phenotypes are the result jointly shaped by the interaction between genotype and environment. Therefore, future research should integrate phenotypic data with genotypic analysis to explore how these observed morphological features are influenced by both genetic factors and environmental conditions. For example, combining genome-wide association studies (GWAS) or transcriptomic data can help identify gene loci or regulatory networks associated with phenotypic indicators such as ENDUI and ENII, and further understand their expression patterns under different environmental conditions. This integrated analysis of phenotype and genotype will contribute to elucidating the molecular mechanisms behind maize kernel morphological variation, providing a scientific foundation and potential targets for directed improvement of kernel traits.

Inspired by the progress in microbial community research (Faust et al., 2012; Peschel et al., 2021), this study proposed the concept of a crop phenotypic interactome network for the first time and successfully constructed a 3D geometric phenotypic interactome network for maize kernels (**Figure 5**). This network systematically integrated 27 different morphological features, revealing the interrelationships and mechanisms of action among these features. The construction of the phenotypic interactome network not only helps identify key phenotypic indicators but also provides new perspectives for elucidating the genetic basis of complex traits. Through the phenotypic interactome network, we found that ENDUI and ENII are central indicators of kernel morphological phenotypes. These two composite indicators occupy a central position in the network, indicating their important roles in the regulation of kernel morphology. Further network analysis revealed the interaction relationships between different morphological features, such as the influence of kernel length, width, and thickness on embryo and endosperm volume (**Figure 5**). These findings provide important clues for a deeper understanding of the genetic mechanisms underlying maize kernel morphological variation. Additionally, the phenotypic interactome network also revealed the association relationships of phenotypic features among different subgroups. The study showed that there were significant differences in certain key indicators among the Mixed, NSS, and TST subgroups. These differences may reflect the regulatory effects of different genetic backgrounds on kernel morphological features.

Although this study significantly improved the accuracy and efficiency of analyzing maize kernel morphological features through a high-throughput phenotyping system based on point cloud data, there are still some limitations. For example, the complexity of data processing and analysis requires high-performance computing resources and professional technical support (Mir et al., 2019; Danilevicz et al., 2021; Kienbaum et al., 2021), which may limit its widespread application in resource-limited situations. Furthermore, the construction and interpretation of new indicators and phenotypic interactome networks still require more experimental evidence to validate their biological significance and application value. Additionally, the research mainly focused on maize kernel morphology and has not been widely validated for its generality in other crops or phenotypic features. These limitations indicate that future efforts are needed to further optimize and extend the application scope of this method.

In summary, the high-throughput phenotyping system based on point cloud data developed in this study, by introducing new morphological phenotypic indicators and constructing a phenotypic interactome network, significantly improved the accuracy and efficiency of analyzing maize kernel morphological features. The surface and internal fine structures of kernels, which are difficult to capture by traditional methods, were precisely recorded, providing rich phenotypic data. This not only provides new targets and strategies for mining genetic diversity and breeding high-yielding and high-quality varieties but also promotes the systematic and refined development of phenotypic research.

## 5 Conclusion

This study successfully developed a high-throughput 3D phenotypic analysis method for maize kernels based on point cloud data, significantly improving the accuracy and efficiency of analyzing kernel morphological features. Through high-resolution point cloud models generated by micro-CT scanning technology, we were able to capture the detailed three-dimensional structure of kernels and propose new morphological phenotypic indicators, such as the ENDUI and the ENII. These new indicators enriched the existing morphological phenotypic data, providing more comprehensive information on kernel morphology. The results showed that the analysis method based on point cloud data not only captured more subtle morphological differences but also revealed the complex interrelationships among different morphological features. Through 3D phenotypic interactome network analysis, we identified key indicators that play crucial roles in morphological regulation, providing new perspectives for the improvement of kernel morphology. Particularly, the study found that the length of the endosperm and embryo played a key role in determining their overall volume, providing important clues for understanding the developmental mechanisms of maize kernels. Furthermore, the analysis of morphological features across different maize subgroups revealed significant morphological differences, which were related to specific genetic backgrounds and adaptive traits. This study not only achieved innovative breakthroughs in technical methods but also provided important insights into the developmental mechanisms of maize kernels, facilitating the development of more precise breeding strategies.

## Data Availability

The raw data supporting the conclusions of this article will be made available by the authors, without undue reservation.
