# Supplementary material for: Precise 3D geometric phenotyping and phenotype interaction network construction of maize kernels
Source: Front Plant Sci. 2025 Apr 8;16:1438594. doi: 10.3389/fpls.2025.1438594 (PMC12011857; doi:10.3389/fpls.2025.1438594)
Supplement: Supplementary file 1 [file Table1.docx]

Supplementary Material

# Supplementary Table

Table S1 Kernel phenotypic trait basic description

| No. | Trait | Abbreviation | Description | Source | Unit |
| --- | --- | --- | --- | --- | --- |
| 1 | Kernel Shape | KS | Ratio of length, width, and thickness of the kernel surface model bounding box | Micro-CT | / |
| 2 | Kernel Volume | KV | Volume of the kernel surface model | Micro-CT | mm^3^ |
| 3 | Kernel Surface Area | KSur | Surface area of the kernel surface model | Micro-CT | mm^2^ |
| 4 | Kernel Specific Surface Area | KSSA | Ratio of surface area to volume | Micro-CT | / |
| 5 | Kernel Sphericity | KSP | Ratio of volume to the volume of the circumscribing sphere | Micro-CT | / |
| 6 | Embryo Volume | EMV | Volume of the embryo surface model | Micro-CT | mm^3^ |
| 7 | Embryo Surface Area | EMS | Surface area of the embryo surface model | Micro-CT | mm^2^ |
| 8 | Embryo Proportion | EMP | Ratio of embryo volume to kernel volume | Micro-CT | / |
| 9 | Endosperm Volume | ENV | Volume of the endosperm surface model | Micro-CT | mm^3^ |
| 10 | Endosperm Surface Area | ENS | Surface area of the endosperm surface model | Micro-CT | mm^2^ |
| 11 | Endosperm Proportion | ENP | Ratio of endosperm volume to kernel volume | Micro-CT | / |
| 12 | Cavity Volume | CV | Volume of the cavity surface model | Micro-CT | mm^3^ |
| 13 | Cavity Proportion | CP | Ratio of cavity volume to kernel volume | Micro-CT | / |
| 14 | 100-Kernel Weight | Hgw | Dry weight of 100 kernels of seed | Kernel for selecting | g |
| 15 | Maximum distance between the embryo and the seed coat | MaD | Maximum distance between the embryo surface and the seed coat surface | Point cloud | mm |
| 16 | Minimum distance between the embryo and the seed coat | MiD | Minimum distance between the embryo surface and the seed coat surface | Point cloud | mm |
| 17 | Embryo Length | EML | Length of the minimum bounding box of the embryo | Point cloud | mm |
| 18 | Embryo Width | EMW | Width of the minimum bounding box of the embryo | Point cloud | mm |
| 19 | Embryo Thickness | EMT | Thickness of the minimum bounding box of the embryo | Point cloud | mm |
| 20 | Endosperm Length | ENL | Length of the minimum bounding box of the endosperm | Point cloud | mm |
| 21 | Endosperm Width | ENW | Width of the minimum bounding box of the endosperm | Point cloud | mm |
| 22 | Endosperm Thickness | ENT | Thickness of the minimum bounding box of the endosperm | Point cloud | mm |
| 23 | Endosperm nutrient density index | ENDI | The density of nutrients in the endosperm | Indicator innovation | / |
| 24 | Endosperm integrity index | ENII | The integrity of the endosperm | Indicator innovation | / |
| 25 | Embryo volume-surface ratio | EMVSR | The relationship between the volume and surface area of the embryo | Indicator innovation | / |
| 26 | Kernel coat tightness index | SCTI | The degree of tightness with which the kernel coat encloses the kernel's internal structure | Indicator innovation | / |
| 27 | Endosperm Density Uniformity Index | ENDUI | The uniformity of endosperm density | Indicator innovation | / |
